# The effectiveness of personalised surveillance and aftercare in breast cancer follow-up: a systematic review

**DOI:** 10.1007/s00520-024-08530-2

**Published:** 2024-05-02

**Authors:** Marissa C. van Maaren, Jolanda C. van Hoeve, Joke C. Korevaar, Marjan van Hezewijk, Ester J. M. Siemerink, Anneke M. Zeillemaker, Anneleen Klaassen-Dekker, Dominique J. P. van Uden, José H. Volders, Constance H. C. Drossaert, Sabine Siesling, on behalf of the NABOR project group, Marissa C. van Maaren, Valesca P. Retel, Bart Knottnerus, Elise van Leeuwen-Stok, Cristina Guerrero-Paez, Jako S. Burgers, Anneke M. Zeillemaker, Marie-Jeanne T. F. D. Vrancken Peeters, Marjan van Hezewijk, Ester J. M. Siemerink, Aafke H. Honkoop, Jeroen Veltman, Ritse Mann, Jannet Wiegersma, Saskia Claassen, Marije L. van der Lee, Cornelia F. van Uden-Kraan, J. C. Korevaar, M. van Korevaar, E. Siemerink, A. M. Zeillemaker, A. Klaassen-Dekker, C. H. C. Drossaert, S. C. Siesling

**Affiliations:** 1 Department of Health Technology and Services Research, Technical Medical Centre, University of Twente, P.O. Box 217, 7500 AE Enschede, the Netherlands; 2 Department of Research and Development, Netherlands Comprehensive Cancer Organisation (IKNL), Utrecht, the Netherlands; 3 Netherlands Institute for Health Services Research (NIVEL), Utrecht, the Netherlands; 4 The Hague University of Applied Sciences, The Hague, the Netherlands; 5 Institution for Radiation Oncology, Arnhem, the Netherlands; 6 Department of Internal Medicine, ZGT, Hengelo, the Netherlands; 7 Department of Surgery, Alrijne Hospital, Leiden, the Netherlands; 8 Department of Surgery, Canisius Wilhelmina Hospital, Nijmegen, the Netherlands; 9 Department of Surgery, Diakonessenhuis, Utrecht, the Netherlands; 10 Department of Psychology, Health & Technology, University of Twente, Enschede, the Netherlands

**Keywords:** Personalised follow-up, Breast cancer, Surveillance, Aftercare

## Abstract

**Purpose:**

Breast cancer follow-up (surveillance and aftercare) varies from one-size-fits-all to more personalised approaches. A systematic review was performed to get insight in existing evidence on (cost-)effectiveness of personalised follow-up.

**Methods:**

PubMed, Scopus and Cochrane were searched between 01–01-2010 and 10–10-2022 (review registered in PROSPERO:CRD42022375770). The inclusion population comprised nonmetastatic breast cancer patients ≥ 18 years, after completing curative treatment. All intervention-control studies studying personalised surveillance and/or aftercare designed for use during the entire follow-up period were included. All review processes including risk of bias assessment were performed by two reviewers. Characteristics of included studies were described.

**Results:**

Overall, 3708 publications were identified, 64 full-text publications were read and 16 were included for data extraction. One study evaluated personalised surveillance. Various personalised aftercare interventions and outcomes were studied. Most common elements included in personalised aftercare plans were treatment summaries (75%), follow-up guidelines (56%), lists of available supportive care resources (38%) and PROs (25%). Control conditions mostly comprised usual care. Four out of seven (57%) studies reported improvements in quality of life following personalisation. Six studies (38%) found no personalisation effect, for multiple outcomes assessed (e.g. distress, satisfaction). One (6.3%) study was judged as low, four (25%) as high risk of bias and 11 (68.8%) as with concerns.

**Conclusion:**

The included studies varied in interventions, measurement instruments and outcomes, making it impossible to draw conclusions on the effectiveness of personalised follow-up. There is a need for a definition of both personalised surveillance and aftercare, whereafter outcomes can be measured according to uniform standards.

## Introduction

While breast cancer incidence has grown over time—up to 2.3 million diagnoses in 2020 worldwide [1]—mortality rates have declined [2, 3]. Consequently, this results in a large number of breast cancer survivors in follow-up care, consisting of two parts: surveillance and aftercare. Surveillance aims to detect asymptomatic locoregional recurrences (LRR) or second primary breast cancers (SPBC) using mammograms and physical examination. The ultimate aim is to curatively treat patients. The ultimate aim is to curatively treat patients. As distant recurrences are in most cases not curable, and the early detection of distant recurrences does not improve prognosis, surveillance does not actively aim to detect these. Aftercare aims to detect diagnosis- or treatment-related side effects and subsequently use interventions to reduce these and improve quality of life (QoL).

Although in some countries guidelines advise to personalise follow-up [4, 5], current guidelines in, for example, the Netherlands and Belgium still advise similar surveillance schedules for all patients [6, 7]. This is probably due to a lack of clinical evidence that adapting surveillance schedules according to risk profiles is effective. However, it is widely known that differences in individual characteristics largely influence LRR and SPBC risks [8]. Furthermore, about 50% of LRRs and 25% of SPBCs are detected by patients themselves due to symptoms, outside of scheduled surveillance visits [9]. Moreover, patients’ beliefs and expectations of surveillance are often not realistic, including the incorrect assumption that surveillance also aims to detect distant metastases and that breast cancer cannot recur in between scheduled visits [10]. Importantly, overall LRR and SPBC risks are low and largely differ among individual patients [11, 12]. This is expected to lead to unnecessary surveillance visits for many patients and perhaps too little visits for specific high-risk patients.

While surveillance often consists of a one-size-fits-all approach, a large variation in aftercare is present, as this is often arranged according to both clinicians’ and patients’ preferences. It depends on the hospital which health care provider is involved (e.g. surgeon, specialised nurse [13]) and whether they make use of prescheduled consultations. Health care providers are also looking for appropriate tools they can use to personalise aftercare [13]. In addition, there are no guidelines on the specific contents of aftercare plans, resulting in unmet supportive care needs regarding fear of cancer recurrence, daily activity and sexual and psychological well-being [14].

Both these arguments and the fact that an increasing number of breast cancer survivors will receive follow-up care, lead to an increasing belief that surveillance and aftercare should be personalised [15–17]. However, clear evidence is needed about the effectiveness of both personalised surveillance and aftercare. The aim of this review was to identify all studies published from 2010 that investigated the effectiveness of personalised surveillance and/or aftercare in curatively treated nonmetastatic breast cancer patients.

## Methods

This review’s protocol has been registered and made available in PROSPERO [18] (CRD42022375770). The Preferred Reporting Items for Systematic Reviews and Meta-Analyses (PRISMA) 2020 checklist [19] was used for transparent reporting (Online Resource 1–2).

### Search strategy

Databases of PubMed, Scopus (including Medline and keywords of Embase) and Cochrane were searched for relevant publications between January 1st, 2010, and November 10th, 2022. Reference lists of relevant reviews were consulted. Studies published before 2010 were excluded, as we expected these to be less relevant for current clinical practice. The full search strategy is shown in Online Resource 3.

### Eligibility criteria

The study population concerned nonmetastatic breast cancer patients ≥ 18 years, starting follow-up after completion of curative treatment (surgery and, if applicable, radiotherapy and/or chemotherapy). Patients could still be treated with endocrine therapy or targeted therapy. We included all intervention-control studies studying personalised surveillance and/or aftercare, including any intervention tailored to a patient’s individual characteristics and designed to be used for the entire follow-up period. Studies that investigated the effectiveness of short-term dietary, physical interventions, cognitive behavioural therapy or psychoeducational interventions, which were not part of a larger intervention designed for use during the entire follow-up period, were therefore excluded. We included studies on all outcomes, except for diagnostic accuracy, feasibility or patient experiences of the intervention only, without evaluating effectiveness.

### Inclusion and data extraction

Two reviewers (JvH, MvM) independently screened and judged all identified studies on title and abstract. In case of doubt or disagreement, the study was included for full-text analysis. Both reviewers independently read the full text and decided on definite inclusion. Discrepancies were extensively discussed and resolved. Co-authors were consulted if necessary. Final data extraction was performed by one reviewer (MvM), and the second reviewer (JvH) was consulted in case of doubts. In case of missing or unclear information on interventions, the study’s first author was consulted. Data on population, intervention, control and outcomes were extracted. As multiple types of interventions and outcomes were studied, the data is presented descriptively.

### Risk of bias

To assess risk of bias (ROB) of included studies, the Cochrane risk of bias tool for randomised (RoB2) [20] or nonrandomised studies (ROBINS-I) [21] were used, whenever applicable. ROB assessment was independently performed by two reviewers, and discrepancies were discussed.

## Results

**Fig. 1:**
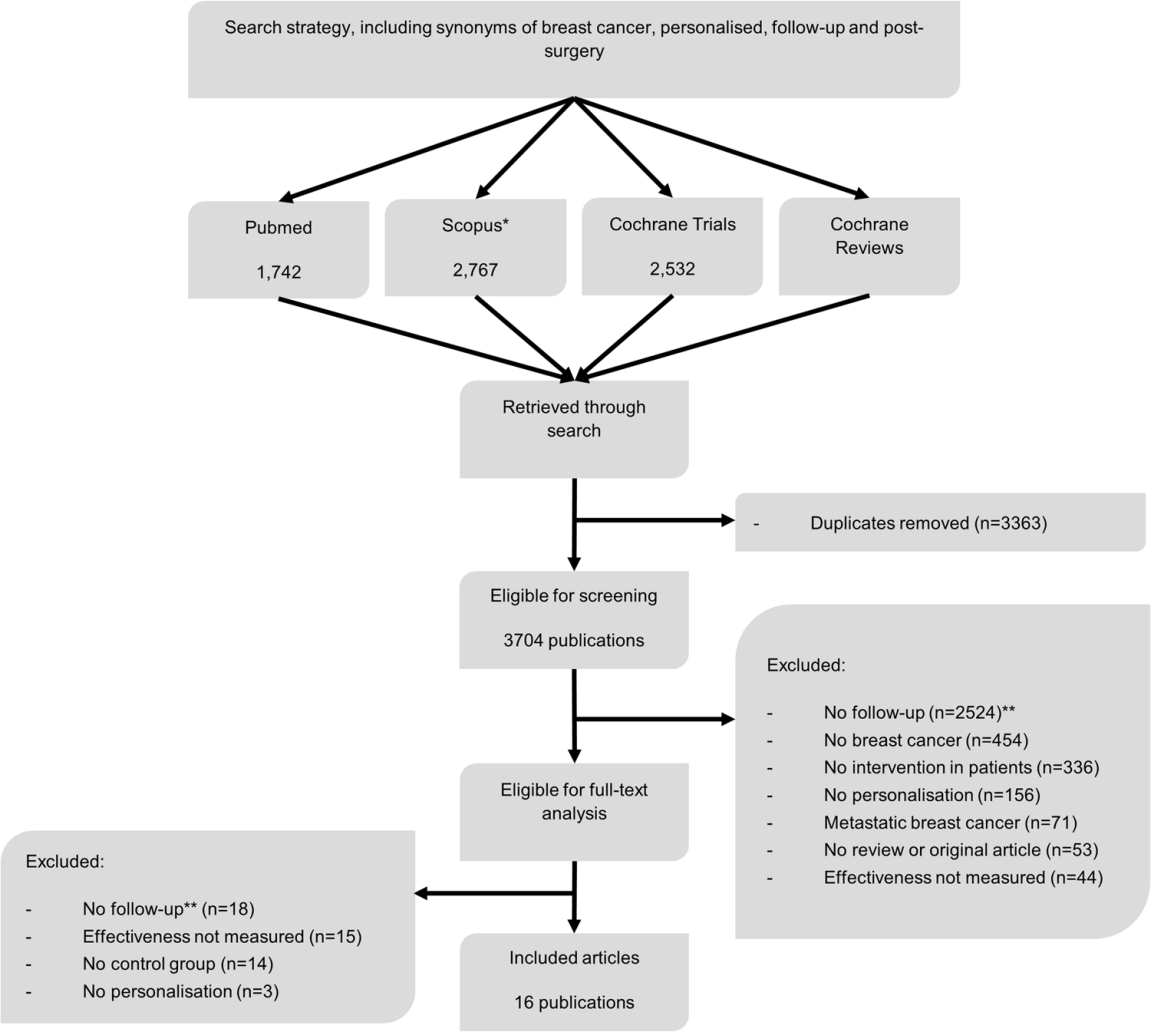
Flow chart of inclusion of publications. *Including Medline and keywords of Embase. **Including studies focusing on short-term dietary, cognitive behavioural, physical or psychoeducational interventions and interventions applied during active treatment

The search strategy yielded 3708 publications. Sixty-four publications were deemed eligible for full-text analysis. After full-text reading, 16 were included for data extraction. Reasons for the exclusion of the other 48 studies were (1) no intervention designed for the entire follow-up, (2) no control group, (3) no effectiveness measured (feasibility studies) and (4) intervention not personalised. Examples of excluded studies are Wallner et al. [22], Haq et al. [23] and Admiraal et al. [24], because they studied feasibility only, did not use a control group, and studied a short-term psychoeducational intervention (so did not personalise the entire follow-up period), respectively. The entire selection is visualised in Fig. 1. The 16 studies finally included are summarised in Table 1. Fifteen studies presented results of randomised controlled trials (RCTs) and one study of a pretest–posttest design. Three studies concerned the same RCT [25–27] but with different outcome assessments (longer follow-up or cost-effectiveness analyses), so were considered different studies in further analysis.

**Table 1:** Detailed overview of included publications with the most important study characteristics

Authors and reference	Type of personalised intervention and design	*N*	Patients	Intervention	Control	Outcome(s)	Conclusion(s)	Risk of bias
Grunfeld et al. [26]	Aftercare, randomised controlled trial	408	Women with early-stage breast cancer who completed primary treatment at least 3 months previously, except for endocrine therapy	An SCP including a personalised treatment summary, a patient version of the Canadian national follow-up guideline, a summary table of the guideline that served as a reminder system and a personalised health care resource list. The documents were reviewed during a 30-min educational session with a nurse who made explicitly clear that surveillance and aftercare were now the responsibility of the primary care physician	All routine surveillance and aftercare were transferred to the patient’s own primary care physician	Primary: change in cancer-specific distress total score at 12 months Secondary: change in total score and subscales of general psychological distress, health-related QoL, patient satisfaction, continuity/coordination of care, the frequency with which patients declined transfer to the primary care physician, the frequency and types of post-discharge visits to the oncologist, awareness of which physician was primarily responsible for follow-up care	No benefit of the SCP on any of the outcomes was found More patients correctly identified the primary care physician as primarily responsible for follow-up care (both surveillance and aftercare)	With concerns
Hershman et al. [28]	Aftercare, randomised controlled trial	141	Women who had a history of stage 0-III breast cancer and were within 6 weeks of completion of initial adjuvant treatment (radiation or chemotherapy)	A National Cancer Institute publication, Facing Forward: life after cancer treatment (a guide for people treated for cancer) plus a personal one-hour meeting with a nurse practitioner and nutritionist to receive a personalised treatment summary, general surveillance recommendations, discussion of risks of side effects and screening and lifestyle recommendations	Only the National Cancer Institute publication, Facing Forward: life after cancer treatment (a guide for people treated for cancer)	Treatment satisfaction, unique and multidimensional aspects of long-term cancer survivorship (impact of cancer), survivor concerns (cancer worry and health worry) after 3 and 6 months	No difference in cancer worry, treatment satisfaction, survivor concerns, depression or impact of cancer. A significant improvement was found in health worry scores at 3 months, but not at 6 months	Low risk
Coyle et al. [25]	Aftercare, a follow-up on Grunfeld et al. [26]	408	See 1	See 1	See 1	Costs and utilities (a standard measure of QoL on a 0 to 1 scale) after 24 months	The SCPs were not cost-effective	With concerns
Rocque et al. [29]	Aftercare, pilot randomised controlled trial	38	Patients diagnosed with stage 0-III breast cancer who completed active treatment and had an email account	Patients first filled in an existing survey on satisfaction with knowledge. Then patients received immediate access of an SCP including all of the recommended Institute of Medicine (IOM) elements (IOM recommends a record of all care received and important disease characteristics, information about the likely course of recovery from acute treatment toxicities as well as the need for ongoing health maintenance or any recommended chemopreventive therapies, including available resources on psychosocial and other practical issues. IOM states that survivorship care needs to be patient-centred and tailored to the patient’s clinical situation and preferences)	Usual care (not further specified). Patients first filled in an existing survey on satisfaction with knowledge. Patients received delayed access to the SCP, after having filled in the survivor knowledge survey (WISDOM-B, study outcome, so after final data collection)	Change in survivor knowledge after 4 weeks	A small difference in survivor knowledge was found, in favour of the intervention group, but it was not significant (the study was not powered to detect a difference of less than 10%)	With concerns
Wheelock et al. [30]	Aftercare, randomised controlled trial	102	Non-metastatic breast cancer who completed active treatment and had access to a computer	SIS-NET: Patients received three breast cancer-related clinic visits with care providers of their choice with additional appointments scheduled later (after the study period) as needed. The intervention included the integration of online health questionnaires at 3-month intervals evaluating symptoms monitored and followed by telephone as necessary by a designated nurse practitioner	Usual care (not further specified). Patients were scheduled for clinic visits based on patient and clinician preference and were invited to complete a routine online health questionnaire before each appointment	Primary: number of days between symptom reporting and remote evaluation and potential management of symptoms after 18 months Secondary: use of health care resources, including the number of breast cancer-related clinic visits, number of total medical appointments and number of laboratory tests and imaging studies	Integration of online health questionnaires with remote review by a nurse practitioner facilitated symptom reporting and may provide a means of convenient symptom assessment, but did not reduce health care resource use	High risk
Boekhout et al. [27]	Aftercare, randomised controlled trial (follow-up on Grunfeld et al. [26])	408	See 1	See 1	See 1	See 1, but with two extensions: - follow-up to 24 months instead of 12 months - additional outcome: adherence to guidelines on follow-up care	No benefit on any of the outcomes was found, including adherence to follow-up guidelines	With concerns
Ruddy et al. [31]	Surveillance and aftercare, randomised phase II trial	100	Non-metastatic female breast cancer patients ≥ 18 years, after treatment	SCP including details on tumour, therapies, providers, screening recommendations and recommended visit frequencies. Patient navigators called/met with patients every 3 months and tried to dissuade patients from scheduling routine surveillance visits within 3 months of each other and made suggestions for visits that could be postponed based on their SCP	Usual care (no SCP or contact with a patient navigator) which was at the discretion of each care provider and not dictated by the study	Primary: the proportion of patients who had at least two breast or chest wall examinations within 30 days of each other without a new related complaint (reflecting potentially unnecessary care) after 12 months Secondary: frequency of visits in general, adherence to standard screening recommendations, QoL, satisfaction with care	SCPs did not lead to statistically significant improvements in patient care. This shows that it is difficult to implement SCPs and patient navigation in a way that meaningfully improves care	With concerns
Kvale et al. [32]	Aftercare, randomised controlled trial	79	Female breast cancer patients diagnosed with stage 0-IIIb breast cancer who were within 1 year of completing active treatment	A POSTCARE intervention: A single coaching encounter utilising motivational interviewing techniques to engage patients in the development of a patient-owned SCP that incorporates a treatment summary, health goals and strategies related to cancer follow-up, surveillance, symptom management and health behaviour	Usual care (not further specified)	QoL, depressive symptoms, self-management, self-efficacy, care coordination at 3 months	The intervention group reported better scores for self-reported health, physical and emotional function roles and demonstrated trends towards improvement in other domains. The intervention group also demonstrated clinically significant improvement in depressive symptoms	With concerns
Maly et al. [33]	Aftercare, randomised controlled trial	219	Female breast cancer patients ≥ 21 years, diagnosed with stage 0-III breast cancer 10 to 24 months earlier, had their last definitive treatment (surgery, chemotherapy, radiation) at least 1 month earlier and were English or Spanish speaking	Receipt of an individually tailored treatment summary and SCP (including specific side-effects of these treatments) and one in-person counselling session with a trained, bilingual, bicultural nurse to review the contents	Usual care (not further specified)	Primary: physician implementation of treatment summaries and SCP Secondary: patient adherence to recommended survivorship up to 12 months and health-related QoL (mental and physical health) at 6 and 12 months	Treatment summaries and SCPs have a significantly positive impact on physician implementation score, but no difference was found for patient adherence and health-related QoL after 12 months	With concerns
Tevaarwerk et al. [34]*	Aftercare, randomised controlled trial	127	Non-metastatic breast cancer patients within 2 years of completing primary treatment	Immediate receipt of an individualised SCP, including relevant breast screening based on patient-specific risk (actual follow-up visits were not tailored), information on bone health and cardiovascular health tailored to patients’ risk, information about treatment in case a patient received it, and specific symptoms and concerns, reported on questionnaires, were covered at regular visits by nurse practitioners or physician assistants	Usual care (not further specified) and delayed receipt of a SCP, after final data collection	Primary: change in survivor knowledge after 12 weeks Secondary: satisfaction with communication and knowledge	A modest improvement in survivor knowledge after 12 weeks was seen in both groups. In the intervention group, this seemed related to repeated administration of the survey rather than receipt of the SCP. No effect on satisfaction	High risk
Ramirez et al. [35]	Aftercare, randomised controlled trial	120	Non-metastatic female Latina primary breast cancer patients ≥ 18 years, deficit in either cancer screening (pap smear/colonoscopy) or a positive comorbidity screening (BMI ≥ 25, diabetes or high glucose level, high blood pressure or current smoker), after treatment	Active support by patient navigators trained in motivational interviewing, providing patients with regular personalised assistance (including phone calls, home visits, transportation assistance, coordination of care), cancer screenings appointments, educational classes, referral to community resources, educational opportunities and help with insurance applications	Patients only received a fact sheet of study services with contact information of patient navigators	QoL after 6 months	Patient navigation led to improved QoL as compared to usual care	With concerns
Riis et al. [36]	Surveillance and aftercare, pilot randomised controlled trial	134	Female postmenopausal nonmetastatic hormonal receptor-positive breast cancer patients ≥ 50 years, after surgery and scheduled for at least 5 years of endocrine therapy	No mandatory consultations. PROs were collected 3 months and used actively as (1) screening tool to assess problems and requirements and (2) as dialogue tool to map symptoms and concerns in order to focus the discussion on what mattered most to the individual patient	Standard follow-up care, including pre-scheduled consultations at 6 monthly intervals for 5 years	Primary: Satisfaction with care, unmet needs after 24 months Secondary: use of consultations, adherence to treatment, self-reported symptoms, functioning, QoL	Individualised follow-up led to equal satisfaction, unmet needs, adherence to treatment and QoL as compared to usual care. However, the number of consultations in the intervention group was lower	With concerns
van der Hout et al. [37]	Aftercare, randomised controlled trial	625	Multiple cancer types (including female breast cancer), both metastatic and non-metastatic patients > 18 years and 3–5 months after curative treatment	Direct access to the web-based eHealth application Oncokompas that supports cancer survivors in self-management by monitoring cancer-generic and tumour-specific symptoms and QoL, providing feedback and information on the scores and a personalised overview of supportive care options	Waitlist – access to Oncokompas after 6 months (after final data collection)	Primary: patient activation after intervention, 3 and 6 months Secondary: QoL, mental adjustment to cancer, supportive care needs, self-efficacy, personal control, perceived efficacy in patient-physician interaction	No significant effect on all outcomes except for a positive effect on QoL and tumour-specific symptom burden	With concerns
Fang et al. [6]	Aftercare, randomised controlled trial	202	Female breast cancer patients ≥ 20 years, diagnosed in the last 5 years. Primary treatment had to be completed with no sign of recurrence, and patients had to speak Mandarin or Taiwanese	Access to a web-based personalised SCP computerised application: Healthy living with breast cancer. Tailored information (e.g. treatments and specific side effects) was presented in seven modules using texts or videos based on literature conducted in Taiwan identifying the needs of women with breast cancer. In addition, a push notification reminded women to take their prescribed medicine, participate in a module, seek remedies for side effects, and the date of next outpatient follow-up	Standard follow-up care. Patients were provided with an educational leaflet with information about timing of follow-up visits, recommended examinations, and special considerations related to years since diagnosis and cancer pathology. They attended routine clinic follow-ups	Primary: unmet needs after 12 months Secondary: fear of recurrence, symptoms, depression, anxiety, QoL	The intervention was effective in decreasing women’s unmet needs after 6 months and decreasing their fear of recurrence after 12 months. QoL was improved. However there was no evidence of an intervention effect on symptom distress, anxiety and depression. NB: women in the intervention group more often received chemotherapy, which can have contributed to the differences	High risk
Rutkowski et al. [38]**	Aftercare, pretest–posttest design with sequential inclusion of intervention and control group	87	Breast cancer survivors discharged from the Wellness Beyond Cancer Programme who received either a standardised or personalised SCP	The personalised aftercare care plan included a treatment summary (i.e. diagnosis, medications received, surgeries, etc.) and follow-up surveillance guidelines specific to breast cancer survivors (e.g. mammogram, breast self-check) with the next follow-up test due date indicated	Similar follow-up surveillance guidelines. The difference with the intervention was the absence of treatment summaries and recommended next follow-up test due dates	Perceived knowledge, perceived efficacy in patient-physician interactions, patient activation pre- and post-reception of the personalised plan	Standardised SCPs offer comparable outcomes on self-efficacy in patient-physician interactions and patient activation to those of personalised plans. Survivors with a personalised care plan reported greater perceived knowledge, however the standardised plans resulted in a significant increase in perceived knowledge from pre to post	High risk
O’Hea et al. [39]	Aftercare, randomised controlled trial	200	Female non-metastatic breast cancer patients ≥ 18 years with an active treatment plan who were alert and oriented and could read and comprehend English at a sixth grade level, and were available for follow-up assessments	Polaris Oncology Survivorship Transition (POST) SCP consisting of 10 components: (1) demographic information, (2) diagnosis and status, (3) medical care team, (4) surgery and therapy details, (5) side effects, (6) dates of recent tests, exams and scans, (7) current medications, (8) barriers, (9) upcoming appointments and/or appointments that needed to be scheduled and (10) libraries of information and references on relevant resource	Standard follow-up care	Physical and emotional symptoms, perceptions of quality of oncology care related to SCPning after 1, 3 and 6 months	In terms of QoL, the present study does not support our theory that the POST would significantly impact QoL in women ending treatment for breast cancer	With concerns

*N*, number of patients included; *SCP*, survivorship care plan; *QoL*, quality of life

*In the paper, it was unclear to what degree the intervention was personalised, and the information was obtained after having contact with the first author

**The first author of the paper was consulted to get more insight in the degree of personalisation of the intervention, which was not entirely clear from the paper. Apart from the personalised treatment summaries, the surveillance was similar for all patients, but personal needs (physical, social, emotional, spiritual and practical) were addressed. Therefore, for this review, we considered only aftercare to be personalised in this study

### Populations

All studies included nonmetastatic breast cancer patients who completed primary treatment, except for adjuvant targeted or endocrine therapy. Two studies focused on very specific populations. The first included Latina breast cancer patients with a deficit in either cancer screening (PAP smear or colonoscopy) or a positive comorbidity screening [35]. The second only included hormonal receptor-positive breast cancer patients ≥ 50 years old undergoing endocrine therapy [36]. One study included multiple cancer types [37].

### Interventions

Interventions could consist of the use of personalised aftercare plans or a combined surveillance and aftercare plans, either or not supplemented with (1) educational or counselling sessions, (2) active support by patient navigators and/or (3) monitoring patient-reported outcomes (PROs).

#### Personalised surveillance

Although nine out of 16 studies included general recommended surveillance guidelines in aftercare plans [25–29, 31, 32, 34, 39], only one study evaluated a form of personalised surveillance [31]: all patients were dissuaded from scheduling routine follow-up visits and received suggestions for visits that could be postponed based on their aftercare plan (including personal tumour and treatment history).

#### Personalised aftercare

Aftercare often concerned a plan containing general information supplemented with personalised information. Of all 16 studies, 12 incorporated personalised treatment summaries [6, 25–29, 31–34, 38, 39], six contained lists of available supportive care resources [25–27, 29, 31, 39], four incorporated PRO measures (PROMS) [30, 34, 36, 37], two contained reminders on next follow-up dates [6, 38], one contained a general guide for people treated for cancer [28] and one contained, additional to a complete overview of individual patient-, tumour- and treatment-related details, information on the medical care team, potential side effects, dates of recent visits, current medications, barriers, upcoming appointments and libraries with further information [39].

Five studies additionally included a 30-min to 1-h educational session with a nurse and/or nutritionist [25–28, 33]. This sometimes consisted of an explanation that follow-up care was now the responsibility of the primary care physician [25–27], and mostly included additional information on the contents of a general aftercare plan. One study included motivational interviewing to engage patients in the development of a patient-owned aftercare plan [32].

One study included active support by patient navigators providing patients personalised assistance including phone calls, home visits, transportation assistance and care coordination, and help with practical things [35]. In three studies, patients regularly met/called with a patient navigator, nurse practitioner or a physician assistant at predefined intervals [31, 34, 35].

Two studies integrated PROMs evaluating symptoms, followed by telephone consults [30] or visits with a nurse or physician assistant [34]. One study had no mandatory consultations, but regularly collected PROs that were used as screening and dialogue tool [36]. Two studies included web-based applications, one supporting cancer survivors in self-management by monitoring symptoms and QoL, providing feedback and personalised supportive care options [37], and one included tailored information on treatment and side effects and included push notifications to remind women to take medicine (e.g. endocrine therapy), participate in a module or seek remedies for side effects [6].

### Controls

Most studies included routine follow-up care as control [6, 25–33, 35, 36, 39], which could differ per hospital and between studies. In one study, this routine follow-up care consisted of outpatient clinic visits which were based on patient and clinician experiences, which could be seen as personalised surveillance. However, this study specifically focused on the use of online health questionnaires that were monitored by a nurse practitioner (intervention) vs. no monitoring (control) and did not describe the potential effectiveness of personalised surveillance [30].

Two studies included elements of the intervention in the control group, like a guide for cancer survivorship [28] or a fact sheet with contact information of patient navigators [35]. One study used similar intervention and control conditions except for personalised treatment summaries and recommended the next surveillance due dates, which were only provided in the intervention group [38].

### Outcomes

**Fig. 2:**
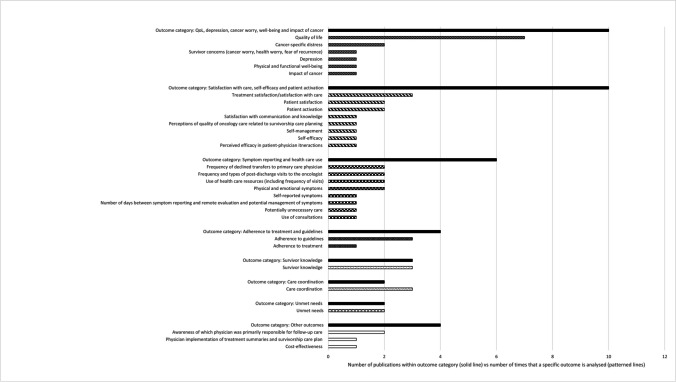
Overview of all outcome(s) (categories) in the 16 included studies. The solid lines indicate the number of studies that analysed a specific outcome category, and the patterned lines indicate the number of times the specific outcome is analysed. The latter numbers do not add up to the numbers in the solid lines, because in one study, multiple outcomes could have been analysed

A detailed overview of all studied outcomes can be found in Table 1. Below, a summary of the most studied outcome categories is given, while Fig. 2 shows all of the specific evaluated outcomes.

#### QoL/depression/cancer worry/well-being/impact of cancer

Ten studies evaluated one of these outcomes. Seven studies evaluated QoL [26, 27, 31–33, 35, 39], and four found a significant positive effect of personalised aftercare on QoL [6, 32, 35, 37]. One of these evaluated active support by patient navigators (differences on subscales ranging from 1.6 to 8.1, all significant except for well-being after 6 months, based on FACT-B/FACT-G questionnaires) [35], one evaluated a web-based eHealth application supporting self-management (summary score difference of 2.3 after 6 months using EORTC QLQ-C30) [37], one evaluated provision of tailored information using a web-based application (summary score difference 6.9 after 12 months, based using WHOQOL-BREF) [6] and one evaluated a coaching encounter to engage patients in the development of a patient-owned aftercare plan (proportion of clinically meaningful improvement in physical role 55 vs.18%, bodily pain 47 vs. 24% and emotional role 42 vs.21% for intervention and control group, respectively, based on SF-36). The latter also found a small significant improvement in depressive symptoms (mean difference of − 1.6 in the intervention group between baseline and 3 months, based on PHQ-9) [32]. Three studies evaluated distress/worries [6, 28, 36], and one found a significant decrease in fear of recurrence (mean difference of − 1.6 after 12 months, based on cancer worry scale) after access to a web-based aftercare plan (high ROB, see the ‘Risk of bias’ section) [6], and one found a significant improvement in health worry after three (mean scores of 2.7 vs. 2.3, respectively, based on ASC), but not after 6 months, for patients who received a personal educational meeting, compared to the control group. They did not find any difference between intervention and control on physical and functional well-being and impact of cancer [28].

#### Satisfaction with care/self-efficacy/patient activation

Ten studies evaluated forms of satisfaction, self-efficacy/self-management or patient activation [26–28, 31, 32, 34, 36–39]. One found a nonsignificant trend towards improvement of self-efficacy and self-management after a coaching encounter to engage patients in a patient-owned aftercare plan [32].

#### Symptom reporting/health care use

Six studies evaluated symptom(s) (reporting) or outcomes related to health care use [26, 27, 30, 31, 36, 39]. One reported a significant positive effect of symptom monitoring using online questionnaires in between standard surveillance visits on symptom reporting (mean of 7.4 vs 3.2 new or changed symptoms within 18 months, respectively), but not on health care resource use [30]. This study had a high ROB (see the ‘Risk of bias’ section). Another study found a significantly lower number of consultations in the intervention group—where PROs were collected and used as screening and dialogue tools—compared to the control group (2.1 vs 4.3 within 2 years, respectively) [36]. All other studies focusing on symptoms, type and/or frequency of care use did not find any significant or clinically relevant differences between intervention and control groups.

#### Adherence to treatment/guidelines

Four studies evaluated treatment/guideline adherence [27, 31, 33, 36], focusing on adherence to recommended visits or adherence to use of endocrine therapy. None of the studies found significant or clinically relevant differences between intervention and control groups.

#### Survivor knowledge

Three studies evaluated survivor knowledge [29, 34, 38], of which two found a significant positive effect of the intervention [34, 38]. One of these evaluated the effect of an individualised aftercare plan but hypothesised that the effect was more related to repeated administration of the survey than receipt of the aftercare plan [34]. The other—which was the only pretest–posttest study included in this review—showed that patients who received a personalised survivorship care plan reported greater perceived knowledge, but that the standardised plans resulted in a significant increase in perceived knowledge from pre to post [38]. Importantly, both of these studies were considered high ROB.

#### Care coordination

Three studies evaluated care coordination [26, 27, 32], but none found significant differences between intervention and control groups. Two of these reported on the same RCT, but with different follow-up times [26, 27]. One study reported a trend towards a positive effect with a mean score of 47.4 vs 35.1 on ‘discussion of survivorship care with primary care physician’ for intervention and control group, respectively [32].

#### Unmet needs

Two studies evaluated unmet needs [6, 36], which were measured by either the Patient Experiences Questionnaire [36] (including open questions on certain procedures that were not offered or concerns that were not discussed with care providers) or the Cancer Survivor Unmet Needs-Chinese Scale [6] (including questions on communication, information, physical/psychological, medical care and communication needs). The latter found a significant decrease in unmet needs after access to a web-based personalised aftercare plan, compared to the control group (mean difference of − 3.6 after 6 months using CaSUN-C, high ROB) [6].

#### Other

Two studies (based on the same RCT) evaluated patients’ awareness of which physician was primarily responsible for follow-up care, as follow-up care was transferred to primary care [26, 27]. Both did not find any significant or relevant effect of a personalised aftercare plan plus an educational session. One study evaluated physician implementation of treatment summaries and a personalised aftercare plan (score based on the number of needs addressed by physicians), and found a significant positive effect (mean difference of 16 (scale of 1–100) after 12 months) [33]. Finally, one study evaluated the cost-effectiveness (based on the same RCT as two other studies that did not report any intervention effects [26, 27]) of a personalised aftercare plan plus an educational session and concluded it was not cost-effective [25].

### Risk of bias

**Fig. 3:**
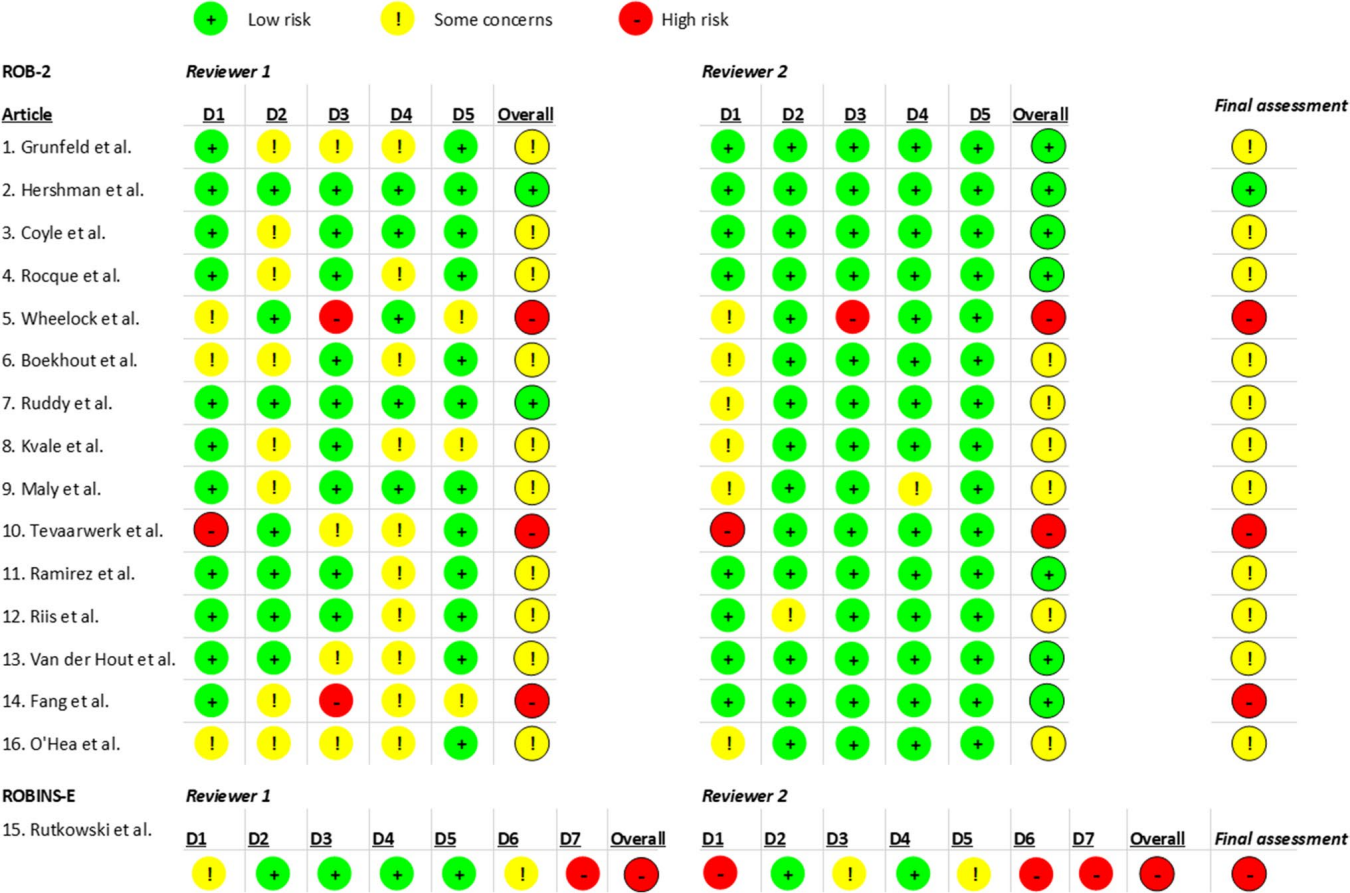
Risk of bias assessment. Upper panel: RoB-2, risk of bias tool for randomised controlled trials; D1, randomisation process; D2, deviations from the intended interventions; D3, missing outcome data; D4, measurement of the outcome; D5, selection of the reported results. Lower panel: ROBINS-E, risk of bias tool for nonrandomised studies; D1, confounding; D2, measurement of the exposure; D3, selection of participants; D4, post-exposure interventions; D5, missing data; D6, measurement of the outcome; D7, selection of the reported results

Of all 16 studies, one study (6.3%) was classified as low [28], four (25%) as high ROB [6, 30, 34, 38] and 11 (68.8%) with concerns [25–27, 29, 31–33, 35–37, 39]. The three studies based on one RCT were all rated as with concerns [25–27]. There were some discrepancies between reviewers which could primarily be explained by different interpretations of signalling questions of domains two and four of RoB2 [20]. This regarded mostly discrepancies between low ROB or having concerns. After careful discussion, the most stringent outcomes were used for the final assessment (Fig. 3, Table 1).

## Discussion

In this review, 16 studies were identified that evaluated the effectiveness of a personalised surveillance and/or aftercare plan in non-metastatic breast cancer patients after curative treatment. A wide range of personalised interventions and different outcomes were studied. Only one study examined a form of personalised surveillance, which did not find any significant or relevant effect on the frequency of visits, adherence to guidelines, QoL and satisfaction with care [31]. Most studies evaluating aftercare plans included individual treatment summaries, overviews of standard follow-up guidelines and/or overviews of available supportive care resources. QoL was most frequently studied, and four out of seven studies found a significantly positive effect of a personalised aftercare intervention. However, most of these studies found small absolute effects. Importantly, only one study was considered to have low ROB, and this study did not find any effect of personalised aftercare. A wide range of other outcomes was studied, with conflicting results. Surprisingly, only one study found a significant effect of personalised aftercare on the outcome category of satisfaction with care/self-efficacy/patient activation, which seems counterintuitive. However, as all studies used different personalised interventions and studied different outcomes using different measurement instruments, it is impossible to compare all studies and to draw conclusions on the effectiveness of personalised follow-up. The fact that three studies were based on one RCT did not affect the conclusions of this review.

Many studies emphasise the need for personalised surveillance [40, 41], but in clinical practice, guidelines still recommend a one-size-fits-all approach [8]. This could be due to many care providers overestimating patients’ recurrence risks [42], or because patients are hesitant about less intensive surveillance [43] due to inadequate risk perceptions, fear of recurrence [44] or unrealistic expectations [10], and could explain that only one of the included studies in this review evaluated a form of personalised surveillance. For both patients and clinicians to get insight in personal risks, a risk prediction tool can be used. INFLUENCE estimates risks of LRR, distant metastases and SPBC [45], and is currently integrated in a decision aid that can be used to personalise surveillance schemes [44]. Recently, this model has been updated to INFLUENCE 3.0 (results not yet published) and is being tested in a large multicentre study on the effectiveness of personalised follow-up, where the model (as part of a decision aid) is used to support the decision regarding the most optimal surveillance scheme [46]. Importantly, the model is based on data from patients that already have been treated for breast cancer, and therefore, the model can explicitly not be used for treatment decision-making. As recurrence rates are generally low [11], it is expected that the frequency of follow-up visits can be reduced for many patients resulting in decreased costs and lower burden on health care [47, 48]. A previous study has already shown that patients are open to the use of risk information in decision-making [43].

The large variety in the type of intervention and outcomes in aftercare suggests that there is a high need for personalisation, but that people are searching for the right way to do so. This is supported by results of several studies, showing large variations in the organisation of aftercare, especially regarding timing, frequency and disciplines of involved care providers [13, 49, 50]. Other studies showed that there are several barriers regarding the integration of PROMs in aftercare [51, 52], which was also evaluated in several of the included studies in this review [30, 34, 36, 37]. It has also been described that promoting engagement and adherence to care plans may lower psychological distress or cancer-related barriers [53]. Studies that evaluated motivational interviewing techniques to increase patient engagement indeed showed significant improvements in QoL [32, 35].

Aftercare is complex and comprises a lot of elements. Ideally, it includes assessment and management of physical and psychosocial effects due to cancer diagnosis and treatment, health promotion and care coordination [54]. In order for patients to get engaged in the management of their own recovery, it is important to empower patients by providing clear information on possible (late) side effects of breast cancer and its treatment—including available self-help and support options—and to give them information on breast awareness (i.e. how to notice potential signs of recurrence in an early state). The relevance of patient empowerment has been acknowledged in literature [55] and has been shown to improve quality of life [56]. In addition, it is crucial for patients to get insight in individual needs. A previous study showed that these individual needs are not always assessed, as only 16.1% asked patients about it [57]. Additionally, many patients have difficulties in expressing their needs [58], and the degree of communication about preferences varies widely between patients with different cultural backgrounds [59]. To support patients to understand their own needs and preferences and to base decisions regarding their health care on it, a patient decision aid or dialogue tool could be used [58, 60], which can form the basis for individual counselling sessions. A prior pilot study showed a newly developed decision aid to have promising effects on shared decision-making, choice evaluation, choice of aftercare and hospital costs, but to substantially increase consultation time [61]. However, one could argue that providing patients with completely individualised aftercare would finally decrease health care use and thus costs. In case a patient timely takes action in case of psychological or physical complaints, or any other concerns, worsening of symptoms and thereby future, more intensive, care use could be prevented. However, this remains to be investigated, as care use might also increase as a result of increased detection of unmet needs. Finally, we can learn from experiences in other cancer types, such as the shared-care survivorship programme for testicular cancer [62] and the Dutch Childhood Oncology Group guideline for follow-up [63].

### Strengths and limitations

To our knowledge, this is the first review that included all published intervention-control studies on the effectiveness of personalised follow-up for breast cancer patients. A broad search strategy was used, ensuring a high level of completeness. Title abstract screening and full-text reading were performed independently by two reviewers, which is described to increase the number of relevant studies identified [64]. ROB assessment was also performed by two independent reviewers, which is crucial since ROB judgements can differ substantially between reviewers, especially regarding interpretation on random sequence generation, blinding of participants and personnel and incomplete reporting [65]. The two reviewers extensively discussed discrepancies, and in case a consensus could not be reached, the most stringent judgement was used for final assessment. Data extraction was performed by one reviewer, which could have resulted in higher error rates [66]. However, as the second reviewer had read all publications’ full text, this reviewer could carefully judge the data extraction on completeness. There were two studies [38, 39] included in this review where both reviewers doubted whether only provision of personalised information on treatments, side effects and/or standard surveillance guidelines (without counselling/educational sessions) could really be considered personalised aftercare. To be complete, these papers were included, also to show the inconsistencies in current practice, confirming the belief that one is still searching for the right way to personalise aftercare.

### Clinical implications

Fifteen out of 16 studies included in this review solely focus on personalised aftercare, and they all include different types of interventions, studied different outcomes and used different measurement instruments. Besides, in some cases, it could be questioned whether the intervention can be called ‘personalised’. This makes it impossible to draw firm conclusions on the effectiveness of the interventions. First, there is need for a definition of personalised surveillance and aftercare. Ideally, surveillance consists of a decision aid including a prediction tool [45] to jointly discuss personalised surveillance schemes. Besides, personalised aftercare should comprise (1) a patient’s needs assessment (e.g. using PROs), (2) information on potential side effects of cancer (treatment) and available care resources and (3) a personalised aftercare plan, including a diagnosis and treatment summary, decisions on organisation of aftercare (e.g. frequency, involved care providers) and signals to seek care for. A dialogue tool could support the shared decision-making process between care professionals and patients of the development of this personalised aftercare plan. Effectiveness can consequently be measured according to uniform information standards such as the ICHOM initiative [67].

## Conclusions and future prospectives

Personalised follow-up varies widely and is not structurally embedded in clinical practice. Therefore, there is still a lack of evidence on its effectiveness. This review shows the current gaps in literature and forms the basis of a large multicentre prospective study on the effectiveness of personalised surveillance and aftercare in breast cancer patients. This prospective study is expected to conquer the problems addressed in this review, and will provide clear evidence on the (cost-)effectiveness of personalised follow-up.

## Data Availability

No datasets were generated or analysed during the current study.

## Abbreviations

ASC: Assessment of Survivor Concerns questionnaire
BMI: Body mass index
CaSUN-C: Cancer Survivor Unmet Needs-Chinese Scale
EORTC QLQ-C30: European Organisation for the Research and Treatment of Cancer Quality of Life questionnaire-30 questions
FACT-B: Functional Assessment of Cancer Therapy-Breast
FACT-G: Functional Assessment of Cancer Therapy-General
ICHOM: International Consortium for Health Outcomes Measurement
LRR: Locoregional recurrence
PAP: Papanicolaou test
PHQ-9: Patient Health Questionnaire-9 questions
PRISMA: Preferred Reporting Items for Systematic Reviews and Meta-Analyses
PROMS: Patient-reported outcome measures
QoL: Quality of life
RCT: Randomised controlled trial
ROB: Risk of bias
RoB2: Risk of bias tool for randomised studies
ROBINS-I: Risk of bias tool for nonrandomised studies
SPBC: Second primary breast cancer
SF-36: Short Form Health Survey-36 questions
WHOQOL-BREF: World Health Organisation Quality Of Life-Brief Version
